# Proposal and Design of Flexible All-Polymer/CIGS Tandem Solar Cell

**DOI:** 10.3390/polym15081823

**Published:** 2023-04-08

**Authors:** Tarek I. Alanazi, Mona El Sabbagh

**Affiliations:** 1 Department of Physics, College of Science, Northern Border University, Arar 73222, Saudi Arabia; tarek.alanazi@nbu.edu.sa; 2 Engineering Physics and Mathematics Department, Faculty of Engineering, Ain Shams University, Cairo 11535, Egypt

**Keywords:** thin film, tandem solar cell, CIGS, all-polymer, TCAD

## Abstract

Tandem solar cells (TSCs) have attracted prodigious attention for their high efficiency, which can surmount the Shockley–Queisser limit for single-junction solar cells. Flexible TSCs are lightweight and cost-effective, and are considered a promising approach for a wide range of applications. In this paper, a numerical model, based on TCAD simulation, is presented to assess the performance of a novel two-terminal (2T) all-polymer/CIGS TSC. To confirm the model, the obtained simulation results were compared with standalone fabricated all-polymer and CIGS single solar cells. Common properties of the polymer and CIGS complementary candidates are their non-toxicity and flexibility. The initial top all-polymer solar cell had a photoactive blend layer (PM7:PIDT), the optical bandgap of which was 1.76 eV, and the initial bottom cell had a photoactive CIGS layer, with a bandgap of 1.15 eV. The simulation was then carried out on the initially connected cells, revealing a power conversion efficiency (PCE) of 16.77%. Next, some optimization techniques were applied to enhance the tandem performance. Upon treating the band alignment, the PCE became 18.57%, while the optimization of polymer and CIGS thicknesses showed the best performance, reflected by a PCE of 22.73%. Moreover, it was found that the condition of current matching did not necessarily meet the maximum PCE condition, signifying the essential role of full optoelectronic simulations. All TCAD simulations were performed via an Atlas device simulator, where the light illumination was AM1.5G. The current study can offer design strategies and effective suggestions for flexible thin-film TSCs for potential applications in wearable electronics.

## 1. Introduction

Recently, solar energy production has become a fast-growing industry and the most abundant form of renewable energy [1]. In this context, photovoltaic (PV) technologies have been developed, paving the way for new materials and structures to boost efficiency while lowering processing costs [2]. First-generation Si-based solar cells have been widely used, and they share about 95% of the PV market, with a record efficiency of more than 26% [3]. In addition, many research efforts have been devoted to alleviating front-side reflectivity issues and enhancing the optoelectronic properties through, for instance, texturization of the silicon wafer by mechanical grooving, utilization of textured optical sheets to improve light trapping, light trapping in silicon nanowires, and hybrid plasmon polaritons [4,5,6,7]. Yet, Si technology has several constraints regarding thick and expensive wafers, in addition to high processing temperatures and expensive fabrication technologies [8]. While numerous efforts have been devoted to lowering the cost of Si solar cells, the power conversion efficiency (PCE) of these proposed architectures still lags behind the conventional high-cost Si cells [9,10,11,12]. On the other hand, novel cost-efficient thin-film solar cells (TFSCs) have been developed to increase the PCE while maintaining reduced prices. Some of the most interesting candidates, in this regard, are CdTe, Cu(In, Ga)Se_2_ (CIGS), polymers, and perovskite photoactive materials, which have been extensively studied thanks to their growing fabrication abilities and accelerated PCEs [13,14,15,16,17,18,19,20,21].

Although the PCE of TFSCs is still lower than that of the conventional Si cell devices, several approaches have recently been employed to achieve competitive efficiency [22,23]. The most crucial technique to increase the efficiency of TFSCs involves designing them in a tandem solar cell (TSC) configuration [24,25]. The most cost-effective way to integrate two subcells is by monolithic 2T TSC, which incorporates a top cell with a wide bandgap and a rear cell with a narrow bandgap. TSCs have been constructed from a wide selection of photoactive materials [21,24,25,26,27,28,29]. Recently, the reported PCEs of monolithic 2T perovskite/Si and perovskite/CIGS TSCs were 31.3% and 24.2%, respectively [30,31]. Furthermore, when considering flexibility, for possible applications in wearable electronics, conventional Si-based cells would not be suitable because of their high-thickness wafers. Candidates such as perovskite, CIGS, and polymer cells are considered favorable in this respect. Thus, to design an eco-friendly and flexible TSC, it is preferred to use materials such as polymers and CIGS instead of perovskite and Si. 

CIGS-based TFSCs and TSCs can be manufactured as flexible devices [32,33]. When incorporating CIGS in TSCs, they are utilized in the bottom subcell. Further, polymer solar cells have attracted research consideration owing to their ease and cheap production as well as their low weight and flexibility. Thus, a suitable polymer for the front subcell can be integrated with CIGS to form a flexible TSC. Of the various types of polymer solar cells (PSCs), all-polymer (in which both acceptors and donors are polymers) solar cells are the most interesting. Some advantages of all-polymer cells include distinguished morphological stability and mechanical endurance [34]. The PCE of all polymer cells has progressively advanced from below 10% to about 18% [35]. Some studies have been conducted to fabricate all-polymer TSCs [36,37,38]. Recently, in [38], PM7:PIDT was selected as the absorber of the top subcell while PM6:PY-IT was utilized as the bottom cell absorber; PIDT and PY-IT are polymerized small molecular acceptors (PSMAs). The TSC configuration achieved a record PCE of 17.87%, with an open-circuit voltage (V_oc_) of 2 V and a short-circuit current (J_sc_) of 11.72 mA/cm^2^.

Eventually, optimizing TSC by engaging trial-and-error experiments is costly and may be futile. Consequently, simulation studies are particularly necessary to provide a more effective and economical approach to optimizing the tandem and recognizing the physical phenomena of cell performance. Different blends have been investigated, and optimization steps have been performed to boost the PCE of the proposed solar cells [39,40,41,42,43]. In addition, many research simulation studies related to CIGS can also be found concerning the impact of defect states, bandgap grading, ultra-thin film absorber, and many other effects, whether for a single-junction CIGS or when incorporating CIGS as a bottom subcell in a TSC [44,45,46,47,48,49]. Regarding all previous analyses, studies on the integration of all-polymer/CIGS TSC by simulation are not present in the literature. 

The primary challenge is achieving efficient charge transport across the interface between the polymer and the CIGS subcells. The use of interfacial layers or buffer layers has been proposed as a solution to this issue for the TSC in general, but optimizing the design and properties of these layers remains an active research area. Another challenge is maintaining the stability of the tandem device over time, particularly with exposure to environmental factors, such as moisture and light. CIGS solar cells now have lifetimes over 25 years due to significant improvements in their stability. In addition, the stability of all-PSCs based on PSMAs show remarkable photostability, and especially endurance to UV light, as advantages [38]. Thus, upon integrating CIGS and all-PSCs, it is expected that the pair would have good stability. Moreover, the cost of CIGS solar cells is estimated to be in line with the cost of other thin-film solar technologies, such as CdTe, and lower than the cost of silicon-based solar cells. In addition, the use of polymer materials in a tandem device can enable low-cost, large-area fabrication techniques such as solution processing, which can reduce manufacturing costs and increase the scalability of the technology.

Based on the aforementioned discussion, all-polymer and CIGS materials have proper complementary absorption comportment. More importantly, owing to the thin film nature of polymers and CIGS solar cells, the produced flexible TSCs have a superior power-to-weight ratio. Thus, the present simulation research proposes a TSC that combines polymers (with an optical bandgap of 1.76 eV) and CIGS (1.15 eV) photoactive materials to be utilized in the top and back subcells, respectively. The bottom cell is a CIGS-based cell, configured as ZnO:Al/ZnO/CdS/CIGS/MO [33], while the top polymer cell is a p-i-n structure composed of PEDOT:PSS/PM7:PIDT/PDINN [38]. To validate the simulation model, performed using the Silvaco TCAD tool [50], a calibration step was performed by simulating the two standalone fabricated all-polymer and CIGS-based solar cells [33,38]. This current simulation study is the first that has integrated all-polymer and CIGS subcells together in a TSC in order to provide a promising way to obtain tandem flexibility and a low cost.

## 2. Simulation Procedure and Device Construction

### 2.1. Silvaco Simulation Platform

**Figure 1:**
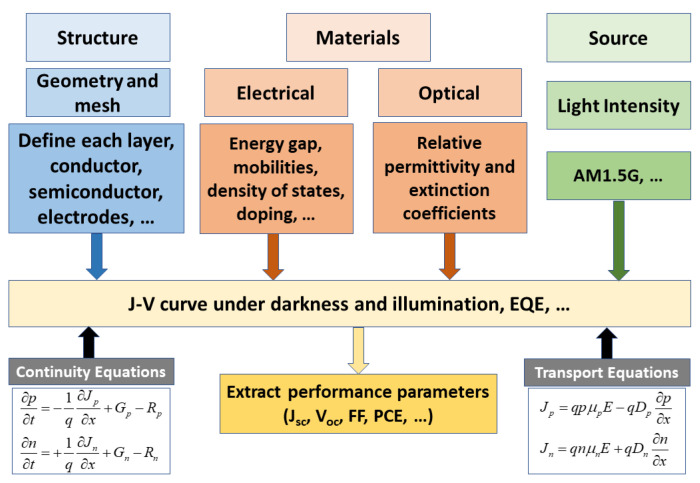
A flowchart displaying the necessary definitions to be prepared before launching the Atlas solver using the current density and continuity equations for simulating a solar cell.

A TCAD Atlas device simulator by Silvaco [50] was employed in the simulations. To simulate a solar cell within the Atlas platform, the cell structure was defined, along with appropriate meshes, in all device regions. In this step, the dimensions of different regions were also specified. The materials of each region were then described with specified doping levels. Physical models were incorporated, such as Shockley–Read–Hall (SRH), Auger and Langevin mechanisms regarding recombination, and Fermi statistics model regarding carrier statistics. In order to obtain the photogeneration rates coupled to the continuity equations, the solar spectra (AM1.5G) were inputted using a BEAM statement within Atlas. Additionally, the extinction constants of all materials involved in the solar cell under investigation were provided. The photogeneration rate was then computed by 2-D ray tracing or 1-D transfer matrix methods. The numerical methods were then determined, and the electron and hole transport equations were solved self-consistently with Poisson’s equation to obtain the desired solution. The current density variation versus voltage (*J-V*) under illumination were obtained, from which the main PV parameters were extracted; J_sc_, V_oc_, fill factor (FF), and PCE. A flowchart summarizing the main simulation steps of the Atlas simulator, along with the principal equations used in the solver, is shown in Figure 1. 

In the following subsections, we describe the implementation technique of the model single junction-based solar cell for both the front and rear subcells. The presented models were based on published experimental data, as discussed herein [33,38]. Next, the calibration of both cells was performed to validate the simulation models involved in the Silvaco simulator.

### 2.2. Top and Bottom Subcell Device Structures and Calibration

The polymer-based device was built starting from an ITO as a front contact (work function = 4.8 eV). A doped PEDOT:PSS layer (p-type, thickness of 30 nm, energy gap *E_g_* = 1.60 eV, electron affinity χ = 3.40 eV, and acceptor doping concentration *N_A_* = 7 × 10^19^ cm^−3^) was used as a hole transport layer (HTL). The light absorber layer was configured by a polymerized small molecule acceptor of PIDT, with a polymer donor of PM7 (undoped, thickness of 100 nm, optical bandgap of 1.76 eV, and χ = 3.74 eV). The resulting highest occupied molecular orbital energy level (HOMO) and the lowest unoccupied molecular orbital energy level (LUMO) of the blend PM7:PIDT were measured as −5.50 and −3.74 eV, respectively [33]. The electron transport layer (ETL) was PDINN (n-type, thickness of 30 nm, *E_g_* = 2.24 eV, χ = 3.78 eV, and donor doping concentration *N_D_* = 1 × 10^19^ cm^−3^). Finally, an Ag contact (work function = 3.72 eV) served as the back contact. 

On the other hand, the standalone CIGS cell was based on CIGS as a thin absorber film (p-type, thickness of 1.6 μm, *E_g_* = 1.15 eV, χ = 4.50 eV, and *N_A_* = 6 × 10^16^ cm^−3^). A Mo layer (work function = 5.5 eV) was in contact with the CIGS layer. The other layers above the CIGS film were CdS buffer (n-type, thickness of 50 nm, *E_g_* = 2.40 eV, χ = 4.25 eV, and *N_D_* = 1 × 10^17^ cm^−3^), i-ZnO (undoped, thickness of 50 nm, *E_g_* = 3.30 eV, and χ = 4.60 eV), and ZnO:Al (n-type, thickness of 200 nm, *E_g_* = 3.30 eV, χ = 4.60 eV, and *N_D_* = 1 × 10^18^ cm^−3^).

**Figure 2:**
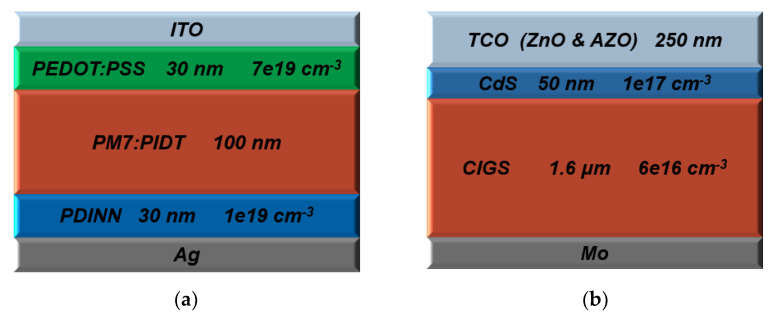
Top and bottom subcell structures, indicating main layer design parameters. (**a**) Polymer cell, (**b**) CIGS cell.

The main factors of the top and rear metal electrodes for the polymer and CIGS cells are listed in Table S1 in the Supplementary Materials file, while the parameters of the defects at the interface’s polymer top cell, as well as trap state parameters for the PM7:PIDT, CIGS, and CdS layers, are introduced in Tables S2 and S3. Schematic cross-section structures of the investigated top and bottom cell devices are demonstrated in Figure 2a and Figure 2b, respectively. Table 1 lists all geometrical and physical factors of the polymer and CIGS cell layers, which were extracted from the recent literature [33,38,51,52,53,54]. In addition, the optical properties regarding the extinction coefficients were obtained from the literature [33,38]. 

**Figure 3:**
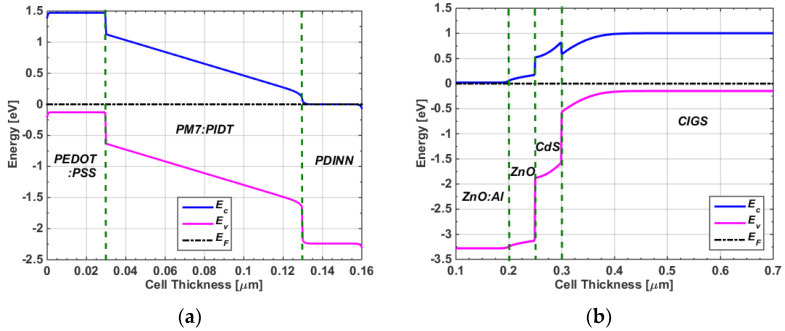
Energy band profiles at dark condition for (**a**) polymer cells (whole distance) and (**b**) CIGS cells (cut up to 0.7 μm).

**Figure 4:**
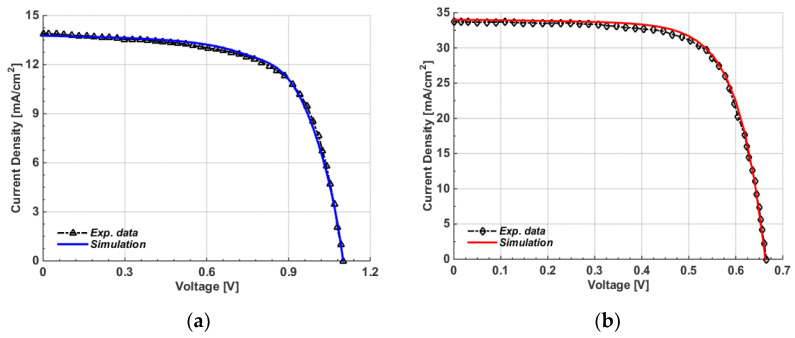
*J-V* characteristics for both simulations and experiments. (**a**) Polymer cells; (**b**) CIGS cells.

**Figure 5:**
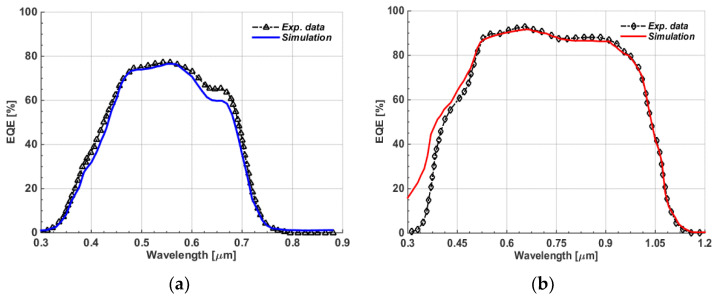
*EQE* characteristics for both simulations and experiments. (**a**) Polymer cells; (**b**) CIGS cells.

After applying all listed parameters and running the simulator, many physical and terminal characteristics were observed. First, Figure 3a,b show the energy band profiles under dark and short-circuit conditions for the front and bottom cells, respectively. Moreover, the *J-V* curves obtained under AM1.5G illumination and experimental data are displayed in Figure 4, where Figure 4a shows the polymer cell curves and Figure 4b illustrates the CIGS cell curves. Additionally, the external quantum efficiency (*EQE*) levels for both the polymer and CIGS cells are shown in Figure 5a and Figure 5b, respectively. Figure 5a demonstrates that the device based on PM7:PIDT exhibited an *EQE* response between 300–750 nm, indicating that this material is well-suited as a photoactive layer for the front cell, capable of absorbing high-energy photons along with its high V_oc_. Further, as shown in Figure 5b, a high *EQE* was achieved in the visible and near-infrared regions of the AM1.5G spectrum, with a peak *EQE* of about 90%. This is due to the fact that CIGS has a narrow bandgap of around 1.15 eV. The *EQE* of CIGS cells drops off rapidly at higher energies, corresponding to the higher-energy photons that are not absorbed by the CIGS material. Further, the PV performance metrics are given in Table 2 for both devices. Based on the presented results, it can be depicted that the experimental results were effectively reproduced by simulation, indicating the suitability of the presented modeling technique for both types of solar cells.

## 3. Results and Discussion

In this section, first, the initial TSC is presented and simulated. Then, a band alignment engineering technique is used to optimize the interface between the ETL/absorber and absorber/HTL of the front polymer subcell. A proper selection of the ETL and HTL materials is then presented. Next, the impact of the thicknesses of both polymers and CIGS light absorbers is investigated to obtain the maximum available PCE. Moreover, the current matching point is investigated, and the results are compared with those acquired from the previous step.

### 3.1. Initial Polymer/CIGS Tandem Design

**Figure 6:**
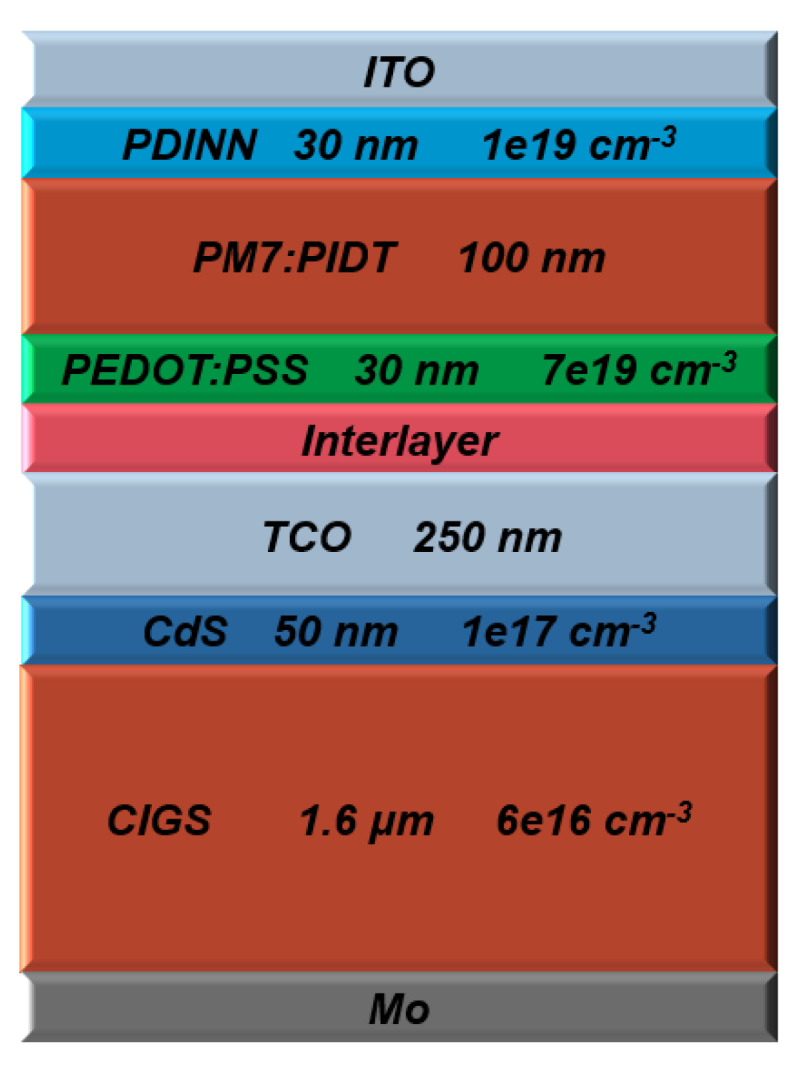
Initial TSC incorporating polymer and CIGS subcells, showing the parameters of the main layer and the interlayer between the subcells.

**Figure 7:**
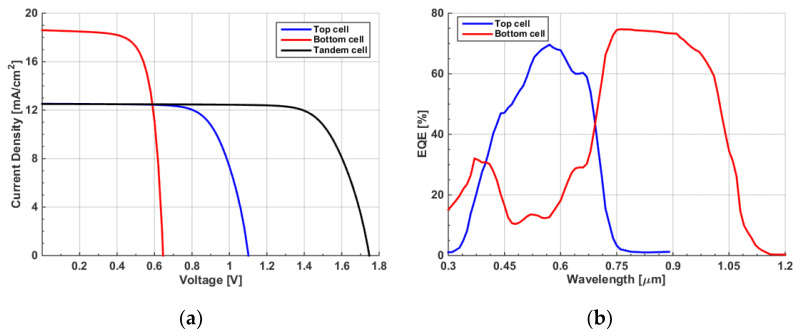
(**a**) Illuminated *J–V* curves of the initial TSC; (**b**) *EQE* spectra.

The initial configuration of the proposed polymer/CIGS TSC is displayed in Figure 6, with the top and back subcells connected via an interlayer, which is modeled by lumped resistance. Practically, this interlayer may be a tunneling junction or an ultrathin metal film such as Ag [22]. After applying the initial settings for both cells, the simulated *J-V* curves of the front, bottom, and tandem cells are exhibited in Figure 7a. It can be inferred from the figure that the tandem J_sc_ had the lowest current of the two subcells. This means that the efficiency was not optimum due to the absence of a current matching condition. On the other hand, V_oc_ was nearly equal to the addition of the corresponding V_oc_ values of the individual subcells, as expected. The *EQE* curves of the subcells are shown in Figure 7b. The short wavelength band nearly below 700 nm is predominantly absorbed by the front cell, while the rear cell absorbs the wavelength range between 700 nm and 1200 nm, which reveals is the presence of proper complementary absorption. However, as the current matching point is not satisfied, the area under the curve of the CIGS cell is higher than that of the all-polymer cell, indicating a higher J_sc_ of the CIGS bottom cell. The extracted output PV parameters are demonstrated in Table 3. As can be observed from the results, the tandem PCE was 16.77%, which was slightly higher than that of the standalone CIGS cell. Thus, the TSC needs to be optimized in order to boost efficiency. In the following subsections, some optimization steps and design recommendations are given.

### 3.2. Optimization of Band Alignment

The main issue regarding heterostructures is the misalignment produced due to the use of different materials. In order to design an efficient TSC, appropriate band alignment between the different constituting layers should be accomplished. Thus, one should design conduction band offsets (CBOs) and valence band offsets (VBOs) and determine which parameters have more dominant influence on the performance. The CBO at the interface of ETL/absorber is defined as CBO = *χ*_abs_ − *χ*_ETL_. Meanwhile, the VBO at the absorber/HTL interface is defined as VBO = (χ_HTL_ + *E_g_*_HTL_) − (χ_abs_ + *E_g,_*_abs_). It has been reported that there are optimum values for CBO and VBO which efficiently support the flow of the photogenerated carriers toward the contacts. These values range from 0 to 0.3 eV and from 0 to 0.2 eV for the CBO and VBO, respectively [55]. As the top cell faces the light from the ETL side, it is important to study the impact of the CBO on the ETL/polymer interface. In addition, we investigated the influence of the VBO at polymer/HTL interface of the top cell.

#### 3.2.1. Impact of CBO of Top Subcell

**Figure 8:**
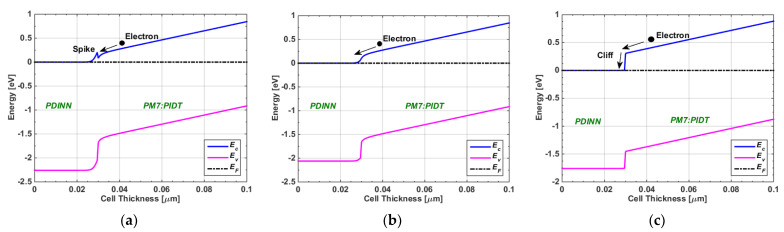
Energy band diagrams of three cases of CBOs at the PDINN/PM7:PIDT interface (for 0.3 eV hole barrier). (**a**) Spike-like band, (**b**) flat band, and (**c**) cliff-like band.

**Figure 9:**
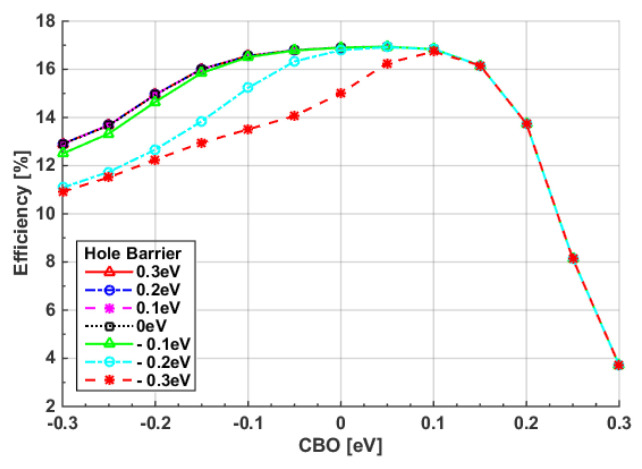
Conversion efficiency, dependency on CBO, at the PDINN/PM7:PIDT interface for different hole barrier values.

In this subsection, we theoretically varied the CBO value while keeping the barrier against the hole transport constant at 0.3 eV. Figure 8 shows the energy band profiles for three cases of CBOs. The first case (see Figure 8a) demonstrated a spike-like band that resulted from a positive CBO. The electron flow was efficient as long as CBO < 0.3 eV; otherwise, a high barrier was produced, which resulted in blocking the carrier transport. For CBO = 0, a flat band was observed (see Figure 8b) in which the charge transport was also proper. Meanwhile, if CBO < 0 (see Figure 8c), a cliff-type band appeared, which did not inhibit charge transport, but increased the interfacial recombination compared to the bulk recombination, resulting in a degraded operation [56]. To provide wider design criteria, we varied the CBO from −0.3 eV to +0.3 eV (which can be adjusted by varying the electron affinity of the ETL) by varying the hole barrier (which can be adjusted by varying the energy gap of the ETL). Figure 9 shows the simulation results regarding the dependency of PCE on the CBO and hole barrier. As is depicted in the figure, the optimum operation for our TSC occurred for CBO values from slightly below 0 to 0.1 eV. It is also recommended to select an ETL material that establishes a hole barrier higher than +0.3 eV.

#### 3.2.2. Impact of VBO of Top Subcell

**Figure 10:**
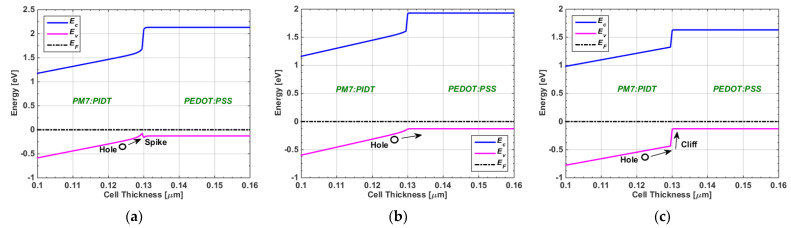
Energy band diagrams of three cases of VBOs at the PM7:PIDT/PEDOT:PSS interface (for 0.3 eV electron barrier). (**a**) spike-like band, (**b**) flat band and (**c**) cliff-like band.

**Figure 11:**
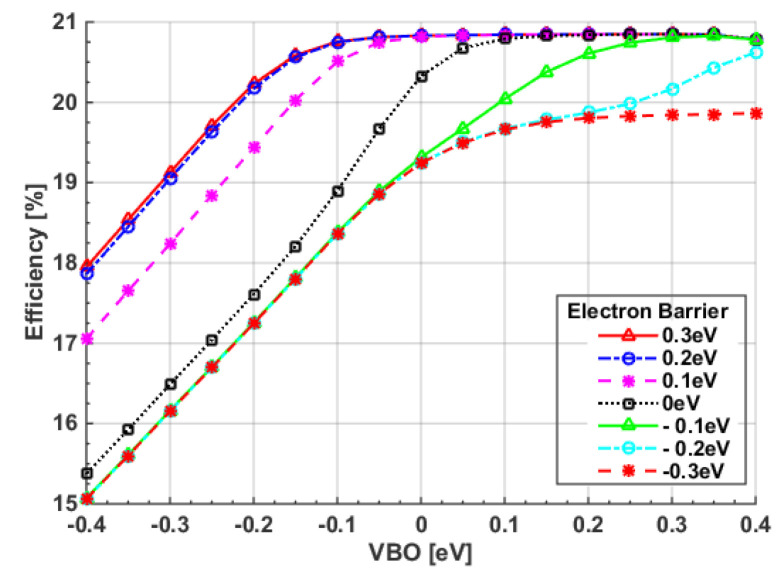
Conversion efficiency dependency on CBO at the PDINN/PM7:PIDT interface for different hole barrier values.

Similarly to the methodology performed above, we also varied the VBO value while keeping the electron barrier at 0.3 eV. Figure 10 illustrates the energy band profiles for three cases of VBOs, namely, the spike type (see Figure 10a), flat band (see Figure 10b), and cliff type (see Figure 10c). In addition, Figure 11 shows the simulation results regarding PCE’s dependency on the VBO and electron barrier. It can be observed that the optimum operation for our TSC occurred for CBO values from slightly below 0 to 0.1 eV. Additionally, it is recommended to select an ETL material that produces a hole barrier higher than +0.3 eV. It can be observed that the optimum operation for our TSC occurred for VBO values from slightly above −0.1 eV to about 0.3 eV. This range of VBO is wider than that of the CBO design. It is also recommended to select an HTL material that yields an electron barrier higher than +0.3 eV.

#### 3.2.3. Optimum ETL and HTL Top Subcell Materials

Based on previous simulations regarding the CBO and VBO, the selection of ETL and HTL materials were carried out according to the design recommendations. Of the possible materials, we found that CdZnS and CBTS could be employed as ETL and HTL, respectively, to achieve the best performance. The technological parameters of the two materials are listed in Table S4 in the Supplementary Materials file [57,58]. When we applied CdZnS as an ETL and CBTS as an HTL, the performance parameters were J_sc_ = 11.40 mA/cm^2^, V_oc_ = 1.91 V, FF = 85.45%, and PCE = 18.57%, demonstrating a significant boost in efficiency.

### 3.3. Optimization of Polymer and CIGS Thickness

**Figure 12:**
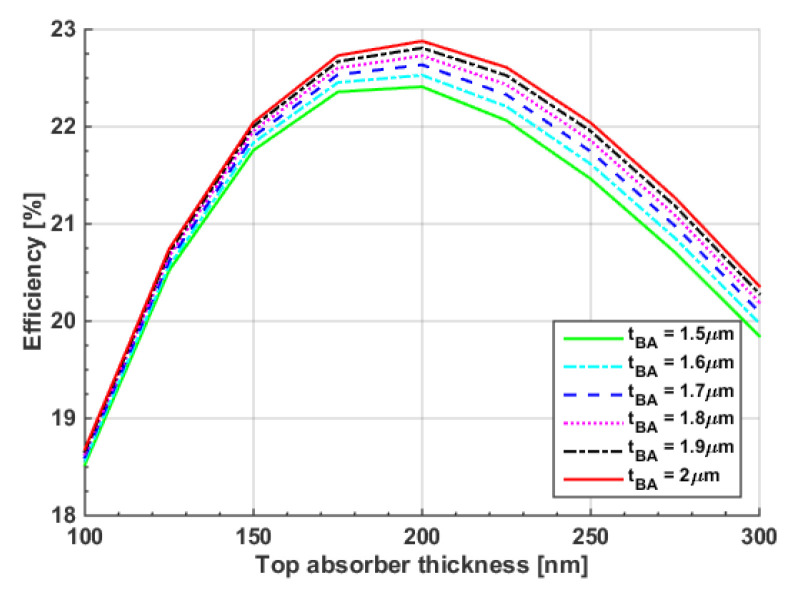
Conversion efficiency dependency on top and bottom absorber thicknesses.

**Figure 13:**
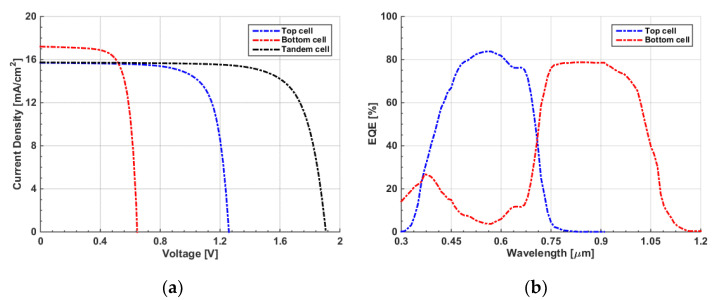
(**a**) Illuminated *J–V* characteristics of top, bottom, tandem. (**b**) *EQE* spectra.

To achieve the maximum PCE from a TSC, a simulation was carried out in which the front and rear absorbers’ thicknesses were simultaneously changed. The contours of the four PV parameters are shown in Figure S1 in the Supplementary Materials file. Figure 12 shows the PCE variation versus the top polymer thickness for different bottom CIGS thicknesses. As can be deduced from the figure, the impact of the polymer thickness has a more significant role than that of the CIGS thickness. It can also be noticed that the maximum efficiency occurred at a polymer thickness of 200 nm. The optimum CIGS thickness value was chosen to be 1.8 μm, with a polymer thickness of 200 nm. Although these thicknesses were wider than the initial values from the fabrication, they were still not far from the technological constraints. In addition, flexibility still held for these thin films. For these selected values, the *J-V* and *EQE* characteristics were plotted as in Figure 13a and Figure 13b, respectively. Again, wavelengths below 700 nm were absorbed by the front subcell, while wavelengths in the range 700 nm–1200 nm were absorbed by the bottom subcell. Although the balanced currents through subcells were disturbed, as the front and rear subcells are not equal, the efficiency of the cells reached the maximum obtainable value, as revealed in Figure 12. This is explained in the following subsection. 

### 3.4. Current Matching Point Simulation

**Figure 14:**
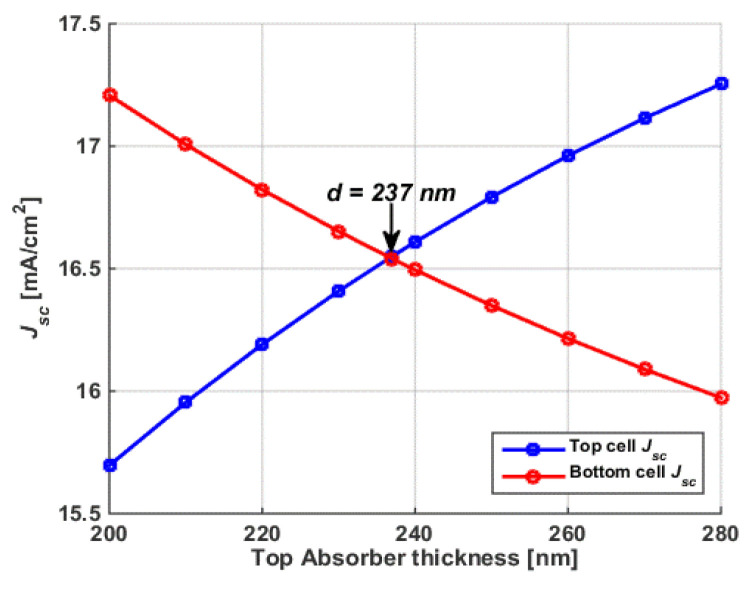
Short-circuit currents of the polymer and CIGS subcells versus thickness of the top subcell absorber.

**Figure 15:**
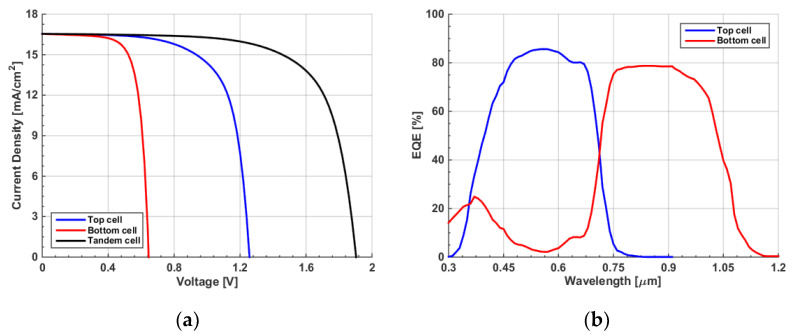
Tandem characteristics under current matching condition. (**a**) *J-V* and (**b**) *EQE*.

It is, notably, essential to achieve balanced currents through subcells of a TSC. However, this does not guarantee obtaining the maximum PCE [59], as is demonstrated herein. In order to achieve the current matching point (CMP), the polymer thickness was varied while maintaining a CIGS thickness of 1.8 μm. The simulation results of the front and bottom J_sc_ are given in Figure 14. From the figure, one can note that the front current rose, and the rear current declined when the polymer thickness was increased because of the intense absorption on thick polymer films, which resulted in lower absorption in the CIGS subcell. The CMP was fulfilled at the intersection point of both J_sc_ curves, which occurred at a polymer thickness of 237 nm. This thickness was remarkably higher than that for the maximum PCE. Thus, it is obvious now that the condition of current matching did not necessarily meet the maximum PCE condition. The *J-V* characteristics of the polymer and CIGS subcells are displayed in Figure 15a, which clearly reveals the verification of the current matching situation. Further, the *EQE* spectra of the two subcells are also shown in Figure 15b, implying perfect complementary absorption between the two subcells.

Finally, Table 4 provides the tandem PV metrics for the current matching and maximum PCE conditions. As is clear from the results, the PCE at the CMP (22.19%) was slightly lower than the PCE at the maximum efficiency condition (22.73%). Although J_sc_ at the CMP (16.55 mA/cm^2^) became higher than that at the maximum PCE (15.74 mA/cm^2^), the fill factor of the former case (70.46%) was much lower than that of the latter case (75.80%). This can be attributed to the increase in series resistance due to wider thicknesses, signifying a higher FF [60]. The overall outcome was that the PCE at CMP was not the maximum available PCE.

Finally, a comparison between the proposed initial polymer/CIGS TSC and the final optimized TSC with the maximum PCE is provided in Figure S2 in the Supplementary Materials file, as well as Table 5. Figure S2 represents the dependence of V_oc_ on light intensity, which provides indications regarding the recombination kinetics of the TSCs under investigation and demonstrates the superiority of the optimized TSC over the initial case. Regarding Table 5, it includes, in addition to our work, some different TSCs and various materials to highlight the state of the art. Some of the listed studies were based on experimental work, while the others were based on simulation using different simulators. It should be noted that higher efficiencies are achieved when incorporating lead-based perovskite in the tandem; however, this places a limitation on the commercialization of this type of tandem due to lead toxicity issues. In [61], a mechanically stacked 4T polymer/CIGS TSC that employed a top polymer (PBDTTPD/PC70BM) subcell was fabricated as a proof of concept for the hybrid tandem PVs. As revealed from the results, the tandem achieved a PCE of 14.50%, although for the standalone CIGS cell, it was 15.70%. This can be attributed to the low V_oc_ obtained by the fullerene-derivative acceptor polymer. On the other hand, our tandem cell showed proper functioning, as its PCE value was higher than that of the bottom cell. Moreover, the top PM7:PIDT cell has already been incorporated into an all-PSC tandem, which has shown satisfactory performance [38], revealing the suitability and feasibility of the chosen top cell to be used in our proposed system. Thus, our work represents the first study which has evaluated and proposed all-PSC/CIGS TSCs, and can inspire further investigations in this field of research.

## 4. Conclusions

In this research work, a 2T polymer/CIGS TSC was proposed. The simulation was carried out in this study using the TCAD device simulator Atlas. All simulations were conducted standardly under AM1.5G light illumination. The design of the TSC is based on an all-polymer top subcell with an optical bandgap of 1.76 eV and a CIGS bottom subcell with a bandgap of 1.15 eV. The two initial standalone cells were based on previous experimental studies. A calibration step was performed first to validate the modeling approach within the simulator. Next, the initial settings of the two cells were tested in an initial TSC, which gave an efficiency of 16.77%, slightly higher than the PCE of the standalone CIGS cell. Thus, to boost efficiency, some design recommendations were addressed and studied. The band alignment treated by manipulating the CBO and VBO of the top cell. The PCE reached 18.57% when optimizing the CBO and VBO by selecting CdZnS and CBTS as the ETL and HTL, respectively. The optimization of polymers and CIGS thicknesses was provided, followed by the investigation of the current matching point. The best performance was obtained for a polymer of 200 nm and a CIGS thickness of 1.8 μm. The PV metrics of the proposed TSC in this optimized case were V_oc_ = 1.91 V, J_sc_ = 15.74 mA/cm^2^, FF = 75.80%, and PCE = 22.73%. 

In conclusion, the presented simulation work represents a novel tandem cell that combines the unique properties of both all-polymer and CIGS solar cells. This offers a potential use for the all-thin-film TSC, which has the potential to be compatible with flexible and lightweight PV technologies, in addition to being eco-friendly. This proposal can inspire further research and development in the field, leading to more efficient and cost-effective solar cells. More methods of optimization should be employed to improve the tandem operation by examining more suitable techniques that can be applied experimentally. 

## Figures and Tables

**Table 1:** Main subcell parameters, used in the simulation, of the different layers.

Parameters	ZnO:Al [51]	ZnO [51]	CdS [52]	CIGS [33,51]	PEDOT:PSS [53]	PM7:PIDT [38]	PDINN [54]
Thickness (nm)	200	50	50	1600	30	100	30
*E_g_* (eV)	3.3	3.3	2.4	1.15	1.6	1.76	2.24
χ (eV)	4.6	4.6	4.25	4.5	3.4	3.74	3.78
*ε_r_*	9	9	10	13.6	3	3	5
μ_n_ (cm^2^/Vs)	100	100	100	300	4.5 × 10^−4^	2.89 × 10^−4^	2 × 10^−6^
μ_p_ (cm^2^/Vs)	25	25	25	30	9.9 × 10^−5^	2.89 × 10^−4^	1 × 10^−3^
*N_C_* (cm^−3^)	2.2 × 10^18^	2.2 × 10^18^	2.2 × 10^18^	2.2 × 10^18^	1 × 10^22^	1 × 10^21^	1 × 10^19^
*N_V_* (cm^−3^)	1.8 × 10^19^	1.8 × 10^19^	1.8 × 10^19^	1.8 × 10^19^	1 × 10^22^	1 × 10^21^	1 × 10^19^
*N_D_* (cm^−3^)	1 × 10^18^	-	1 × 10^17^	-	-	-	1 × 10^19^
*N_A_* (cm^−3^)	-	-	-	6 × 10^16^	7 × 10^19^	-	-

**Table 2:** Comparison between measured and simulated optoelectronic parameters of PM7:PIDT and CIGS-based solar cells.

PV Parameters		V_oc_ (V)	J_sc_ (mA/cm^2^)	FF (%)	PCE (%)
PM7:PIDT Cell	Exp. Data [38]	1.10 ± 0.01	14.0 ± 0.3	65.3 ± 1.2	10.1 ± 0.3
	Simulation	1.10	13.76	66.77	10.11
CIGS Cell	Exp. Data [33]	0.665	33.75	71.30	16.01
	Simulation	0.663	34	71.26	16.05

**Table 3:** PV parameters of initial polymer/CIGS tandem cell.

PV Parameters	V_oc_ (V)	J_sc_ (mA/cm^2^)	FF (%)	PCE (%)
Top cell	1.10	12.52	71.56	9.87
Bottom cell	0.65	18.60	72.63	8.71
Tandem cell	1.75	12.51	76.75	16.77

**Table 4:** PV parameters of polymer/CIGS TSCs at maximum PCE and current matching conditions.

	Maximum PCE Condition				Current Matching Condition			
PV Parameters	V_oc_ (V)	J_sc_ (mA/cm^2^)	FF (%)	PCE (%)	V_oc_ (V)	J_sc_ (mA/cm^2^)	FF (%)	PCE (%)
Top cell	1.26	15.70	74.99	14.82	1.26	16.55	69.37	14.43
Bottom cell	0.65	17.21	73.28	8.15	0.65	16.55	73.37	7.83
Tandem cell	1.91	15.74	75.80	22.73	1.90	16.55	70.46	22.19

**Table 5:** State of the art comparison between typical PV factors of different TSCs reported in the literature.

Top Subcell (*E_g_* (eV))	Bottom Subcell (*E_g_* (eV))	V_oc_ (V)	J_sc_ (mA/cm^2^)	FF (%)	PCE (%)	Notes	Ref.
Perovskite (1.77)	Organic (1.41)	1.902	13.05	83.1	20.60	Experimental	[26]
Pb-free Perovskite (1.61)	Organic (1.33)	1.580	13.29	82.15	17.46	Simulation with SETFOS	[21]
Perovskite (1.76)	Perovskite (1.08)	2.03	16.5	79.90	26.70	Experimental	[29]
Perovskite (1.75)	Perovskite (1.25)	2.047	18.30	86.23	32.30	Simulation with SCAPS	[25]
Perovskite (1.68)	CIGS (1.10)	1.77	18.8	71.2	24.20	Experimental	[29]
Perovskite (1.55)	CIGS (1.10)	1.23	28	80.71	27.74	Simulation with SCAPS	[48]
Polymer (≈1.70)	CIGS (≈1.10)	1.63	NA	NA	14.50	Experimental	[61]
All-Polymer (1.67)	CIGS (1.15)	1.75	12.51	76.75	16.77	Simulation with TCAD Silvaco (Initial)	This work
All-Polymer (1.67)	CIGS (1.15)	1.90	16.55	70.46	22.19	Simulation with TCAD Silvaco (Optimized)	This work

## Data Availability

No new data were created or analyzed in this study. Data sharing does not apply to this article.

## Supplementary Materials

The following supporting information can be downloaded at: https://www.mdpi.com/article/10.3390/polym15081823/s1, Figure S1: Performance parameters dependency on top and bottom absorber thicknesses; Figure S2: Dependence of V_oc_ on the incident light intensity; Table S1: Main factors of the top and back metal contacts for polymer and CIGS cells; Table S2: Defects parameters at the interfaces of polymer cell; Table S3: Trap state parameters for the PM7:PIDT, CIGS, and CdS layers used in device simulation; Table S4: Basic parameters of CdZnS and CBTS layers. References [62,63,64,65] are cited in the Supplementary Materials.
