# Supplementary material for: Proposal and Design of Flexible All-Polymer/CIGS Tandem Solar Cell
Source: Polymers (Basel). 2023 Apr 8;15(8):1823. doi: 10.3390/polym15081823 (PMC10142275; doi:10.3390/polym15081823)
Supplement: Supplementary file 1 [file polymers-15-01823-s001.zip › polymers-2288074-supplementary.pdf]

Supplementary Material

Proposal and Design of Flexible All-Polymer/CIGS Tandem Solar Cell

Tarek I. Alanazi and Mona El Sabbagh

Table S1. Main factors of the top and back metal contacts for polymer and CIGS cells.

	Contact	Material	Work function (eV)	Electron surface recombination velocity (cm/s)	Hole surface recombination velocity (cm/s)
Polymer cell	Front metal	ITO	4.8	1×10^{7}	1×10^{6}
	Back metal	Ag	3.72	1×10^{6}	1×10^{7}
CIGS cell	Front metal	ITO	4.7	1×10^{7}	1×10^{6}
	Back metal	Mo	5.5	1×10^{6}	1×10^{7}

Table S2. Defects parameters at the interfaces of polymer cell.

	**PEDOT:PSS/Polymer**	**Polymer/PDINN**
Defect type	Neutral	Neutral
Electron capture cross section (cm^2)	3.2×10^{-16}	1×10^{-15}
Hole capture cross section (cm^2)	1×10^{-15}	8×10^{-16}
Energetic distribution	Single	Single
Energy level with respect to the highest E_v (eV)	0.6	0.6
Total density (cm^{-2})	2.5×10^{11}	1×10^{11}

Table S3. Trap states parameters for the PM7:PIDT, CIGS and CdS layers used in device simulation.

**Parameter**	**PM7:PIDT**	**CIGS**	**CdS**
Defect type	Donor	Donor	Acceptor
Energetic distribution	Single	Single	Single
Capture cross section (cm^2)	1×10^{-15}	1×10^{-15}	1×10^{-15}
Energy level (eV)	0.88 (above E_v)	0.57 (above E_v)	1.2 (above E_v)
Total density (N_t) (cm^{-3})	1×10^{10}	1×10^{13}	1×10^{12}

Table S4. Basic parameters of CdZnS and CBTS layers.

**Parameters**	**CdZnS [S1]**	**CBTS [S2]**
Thickness (nm)	30	30
Energy gap (eV)	3.18	1.9
Electron affinity (eV)	3.71	3.6
Relative permittivity	10	5.4
Electron mobility (cm^2/Vs)	340	30
Hole mobility (cm^2/Vs)	50	10
CB effective density of states (cm^{-3})	2.5×10^{18}	2.2×10^{18}
VB effective density of states (cm^{-3})	2.5×10^{19}	1.8×10^{19}
Shallow donor density N_D (cm^{-3})	5×10^{18}	-
Shallow acceptor density N_A (cm^{-3})	-	5×10^{18}

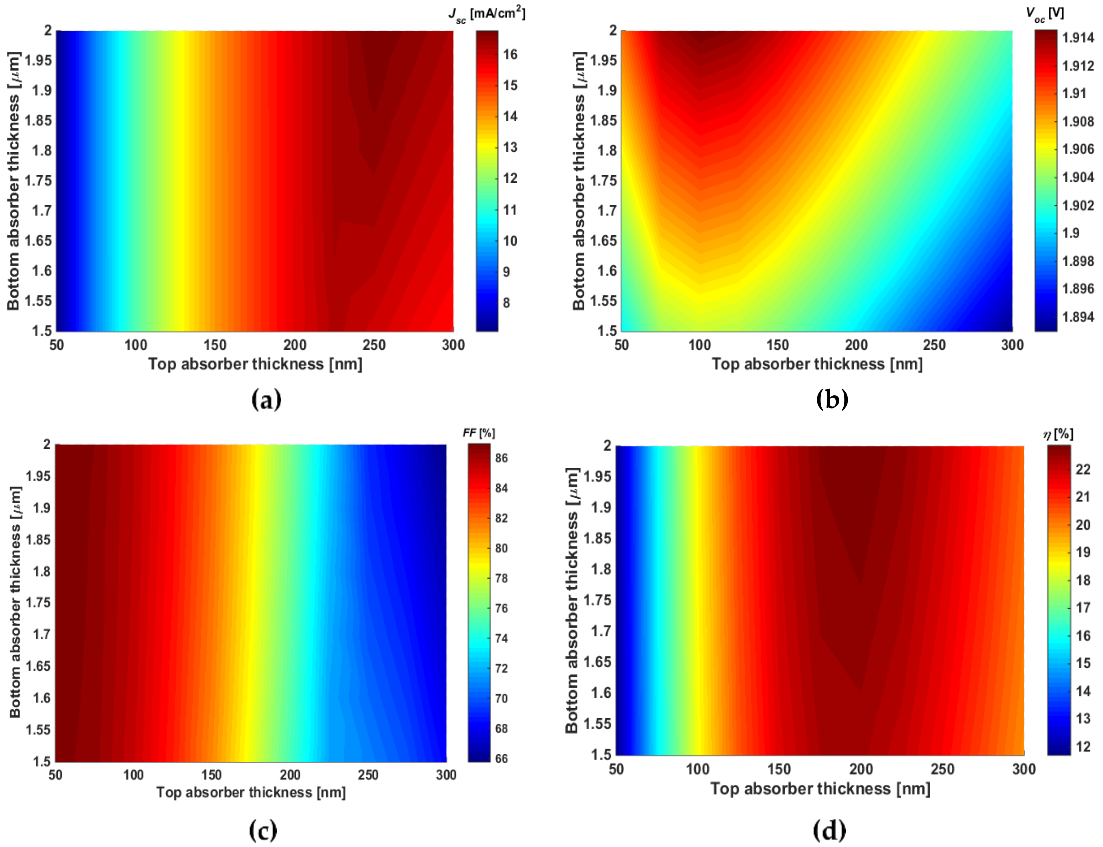

Figure S1. Performance parameters dependency on top and bottom absorber thicknesses.

Regarding the dependence of V_{oc} on the intensity (I), it can be given as [S3],

$$V_{oc} = \varepsilon V_T \, ln(I) + \text{constant} \quad \text{(S1)}$$

Where V_T is the thermal voltage and ε is the ideality factor [S4]. It was realized that the solar cell displays extra SRH recombination rates when ε is more than one [S5, S6]. The values of ε were determined as 1.973 and 1.823 for initial and optimized TSCs, respectively as shown in Figure S2.

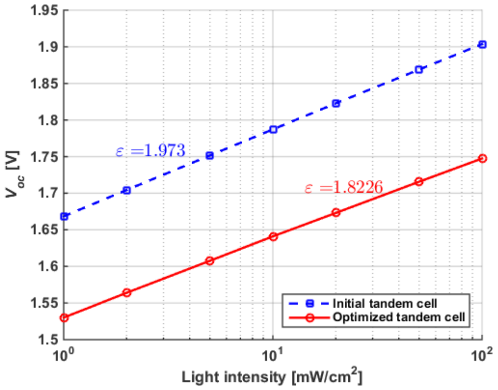

Figure S2. Dependence of V_{oc} on the incident light intensity.

References

[S1] Salem, M.S.; Shaker, A.; Othman, M.S.; Al-Bagawia, A.H.; Fedawy, M.; Aleid, G.M. Numerical analysis and design of high performance HTL-free antimony sulfide solar cells by SCAPS-1D. *Optical Materials* **2022**, *123*, 111880.

[S2] Hossain, M.K.; Rubel, M.H.K.; Toki, G.I.; Alam, I.; Rahman, M.F.; Bencherif, H. Effect of various electron and hole transport layers on the performance of $CsPbI_3$-based perovskite solar cells: A numerical investigation in DFT, SCAPS-1D, and wxAMPS frameworks. *ACS Omega* **2022**, *7*(47), 43210-43230.

[S3] Leong, W.L.; Ooi, Z.E.; Sabba, D.; Yi, C.; Zakeeruddin, S.M.; Graetzel, M.; Gordon, J.M.; Katz, E.A.; Mathews, N. Identifying Fundamental Limitations in Halide Perovskite Solar Cells, *Adv. Mater.* **2016**, 28, 2439–2445.

[S4] Zhang, H.; Cheng, J.; Lin, F.; He, H.; Mao, J.; Wong, K.S.; Jen, A.K.Y.; Choy, W.C.H. Pinhole-free and surface-nanostructured niox film by room-Temperature solution process for high-performance flexible perovskite solar cells with good stability and reproducibility, *ACS Nano* **2016**, 10, 1503–1511.

[S5] Kyaw, A.K.K.; Wang, D.H.; Gupta, V.; Leong, W.L.; Ke, L.; Bazan, G.C.; Heeger, A.J. Intensity dependence of current–voltage characteristics and recombination in high-efficiency solution-processed small-molecule solar cells. *ACS Nano* **2013**, *7*(5), 4569-4577.

[S6] Salem, M.S.; Shaker, A.; Abouelatta, M.; Alanazi, A.; Al-Dhlan, K.A.; Almurayziq, T.S. Numerical analysis of hole transport layer-free antimony selenide solar cells: Possible routes for efficiency promotion. *Optical Materials* **2022**, *129*, 112473.
